# Special Issue “Emotion Intelligence Based on Smart Sensing”

**DOI:** 10.3390/s23031098

**Published:** 2023-01-18

**Authors:** Sung Park, Mincheol Whang

**Affiliations:** 1 Department of Emotion Engineering, Sangmyung University, Seoul 03016, Republic of Korea; 2 Department of Human-Centered Artificial Intelligence, Sangmyung University, Seoul 03016, Republic of Korea

Emotional intelligence is essential to maintaining human relationships in communities, organizations, and societies. By definition, emotional intelligence refers to how well emotion is recognized and expressed. The level of emotional intelligence of an AI is mainly determined by its ability to accurately and reliably recognize its human counterpart; that is, all next-generation AI devices and services involving VR, AR, and social robots are able to quantitatively track and recognize emotion in real-time during an interaction with a human.

Emotion has been quantified by sensing facial expressions, gestures, and physiological signals such as EEG, ECG, and EDA. In addition, emotion could be more accurately recognized by considering the emotional context, including spatiotemporal variability, the congruency of implicit and explicit responses, the consistency of human action, and human relationships in society. Human emotion includes not only short-term but also long-term responses to patterns and trends in daily life. Lab studies with the aim of sensing emotion should extend to smart sensing, which monitors and tracks emotional variation with a predictable pattern.

This Special Issue explores empirical studies of emotional mechanisms, qualitative and quantitative measurements of emotion, the recognition of emotional contexts, and the application of emotion. Fourteen papers were accepted for publication in this Special Issue entitled “Emotion Intelligence Based on Smart Sensing”, which includes papers ranging from lab-based studies aimed at understanding emotional mechanisms to applying emotion recognition in the real world (e.g., in driving, games, education, and virtual avatars). They are summarized below.

The review paper in [1] presents a detailed analysis of over 600 papers related to sensors and methods to understand affective-, emotional-, and physiological-state recognition. Facial action coding and facial expression analysis are long-studied fields, as represented by four articles in our SI. While facial recognition systems in the real world (i.e., in an uncontrolled environment) have evolved with performance improvements, [2] proposed a multi-spectral facial recognition system that overcomes the limitation of a single spectral band in the visible spectrum. The multi-spectral facial recognition system is robust to occlusions (e.g., fog or plastic materials) and low- or no-light environments. The authors of [2] achieved 99.5% (pose variation) and 99.6% (expression variation) Rank-1 scores in the TUFTS multi-spectral database. As AI technology evolves rapidly, so does the facial expression recognition algorithm. The authors of [3] proposed a multi-depth network that classifies facial expressions by being fed reinforced features. A multi-rate-based 3D convolutional neural network (CNN) built on a multi-rate signal process scheme was suggested, and they achieved 96.23% accuracy with the CK+ dataset.

Building an emotionally intelligent system requires a better understanding of human facial expression characteristics. The authors of [4] investigated the differences in the intensity of facial expressions between older (n = 56) and younger adults (n = 113). The participants’ facial expressions were elicited using facial expression stimuli. The results indicated that the older adults strongly expressed some negative and neutral emotions. In addition, older adults used more facial muscles than younger adults across emotions. Human facial expressions include facial micromovements, which provide insights into fake expressions. The authors of [5] investigated the characteristics of real and fake facial expressions representing emotions by analyzing participants’ facial micromovements. The results indicated significant differences in the micromovement feature variables between the real and fake expression conditions. The differences varied according to facial regions as a function of emotions.

This issue also includes a speech-emotion-recognition study [6] that proposed a multi-path and group-loss-based network (MPGLN) for emotion recognition to support multi-domain adaptation. The authors proposed a model that includes a bidirectional long short-term memory-based temporal feature generator and a transferred feature extractor from the pre-trained VGG-like audio classification model (VGGish). The model learns simultaneously based on multiple losses according to the association of emotion labels in the discrete and dimensional models.

The simultaneous activation of brain regions (i.e., brain connection features) is an essential mechanism of brain activity in emotion recognition, and this issue presents three EEG-based studies that advance such science. The authors of [7] investigated the relationship between brain connectivity (strength and directionality) and eye movement features (left and right pupils, saccades, and fixations) when participants (n = 47) viewed emotion-eliciting content. They found that the connectivity eigenvalues of the long-distance prefrontal lobe, temporal lobe, parietal lobe, and center were related to cognitive activity involving high valance. In addition, saccade movement was correlated with long-distance occipital–frontal connectivity. The authors of [8] investigated model-free functional connectivity metrics along with deep learning to efficiently classify human cognitive workload. They achieved state-of-the-art multi-class classification accuracy of 80.87% using a combination of MI (Mutual Information) and CNN, followed by 75.88% using a combination of PLV (Phase Locking Value) and CNN (at), and 71.87% using MI with LSTM. The authors of [9] constructed a learning emotion EEG dataset (LE-EEG) which captures physiological signals reflecting the emotions of boredom, neutrality, and engagement during learning, and proposed an EEG emotion classification network based on attention fusion (ECN-AF). On the LE-EEG dataset, the proposed model achieved the highest accuracy of 95.87%, demonstrating a 21.49% increase compared to the baseline models.

Biological hormones are relatively less explored, but could provide insights into negative emotions such as fear or panic. The authors of [10] investigated catecholamines, which are hormones released in the body in response to physical or emotional stress. They analyzed physiological signals in reference to catecholamine through an experimental task whereby 21 female volunteers received audiovisual stimuli through an immersive virtual-reality environment.

The essence of emotional intelligence overlaps with empathy, a psychological construct. A system that analyzes whether a human is empathizing is paramount. The authors of [11] suggested a non-contact method for measuring empathy by evaluating the synchronization of facial micro-movements between consumers and people in the media. Their study shows that the non-contact ballistocardiography (BCG) method can be complementary to subjective empathy scales.

Finally, this issue also extends to studies applicable to the real world (e.g., in driving, games, and virtual agents). The authors of [12] proposed a data collection system that collects multimodal emotion datasets during real-world driving. The proposed system includes a self-reportable HMI application into which a driver directly inputs their current emotion state. To demonstrate the collected dataset’s validity, the paper provides case studies for statistical analysis, driver face detection, and personalized driver emotion recognition. The authors of [13] used electrocardiograms (ECGs) to investigate heart rate variability (HRV) parameters that can quantitatively characterize game addiction. The participants played the game *League of Legends*, and the experimenter performed ECG measurements during the game at various window sizes and specific events. The correlation and factor analyses were used to find the most effective parameters. The most accurate set of parameters was found to be pNNI20, RMSSD, and LF within 30 s after the “being killed” event. The authors of [14] investigated elements that may affect a the participant’s social perceptions (similarity, familiarity, attraction, liking, and involvement) of customized virtual avatars engineered considering the user’s facial characteristics. The results indicated that participants felt that the avatar that embodied their habitual expressions was more similar to them than the avatar that did not.
